# Integration of patient and public involvement in a doctoral research study using the research cycle

**DOI:** 10.1186/s40900-024-00620-z

**Published:** 2024-08-09

**Authors:** Helen Pearson, Carol Bell, Karl Cox, Catherine Kayum, Leona Knox, Faith Gibson, Michelle Myall, Anne-Sophie Darlington, Emma Potter, Nicholas Bird

**Affiliations:** 1 School of Health Sciences, University of Southampton, Southampton, UK; 2 The Oak Centre for Children and Young People, The Royal Marsden NHS Foundation Trust, Sutton, Surrey UK; 3 Member of the REDMAPP Patient Public Involvement Group, London, UK; 4 Solving Kids’ Cancer UK, London, UK; 5 Centre for Outcomes and Experience Research in Children’s Health, Illness and Disability (ORCHID), Great Ormond Street Hospital for Children NHS Foundation Trust, London, UK; 6 School of Health Sciences, University of Surrey, Guildford, Surrey UK

**Keywords:** Childhood cancer, Co-design, Co-production, Decision-making, Inclusion, Paediatric, Patient public involvement, Parents

## Abstract

**Background:**

Patient and public involvement (PPI) in research is widely acknowledged as essential to achieving successful and impactful research. Despite this acknowledgement, there are limited reports on how to approach and apply meaningful PPI throughout the research cycle and how to address challenges for researchers such as doctoral students, particularly when undertaking research on sensitive topics. This paper provides insights and examples for researchers new to PPI, on the impact of active PPI and recommendations for building and developing a PPI group in a paediatric focused doctoral research study with bereaved parents and carers.

**Methods:**

PPI was informed by the research cycle. The GRIPP2 short-form checklist was used to report PPI. The research was funded by the National Institute for Health and Care Research.

**Results:**

PPI enhanced the research through input into the study design, recruitment, co-design of the study website and branding; and ethics amendments to increase participation in response to the COVID-19 pandemic. The literature review was extended to incorporate a PPI consultation phase and members contributed to data analysis. A flexible approach enabled involvement to develop iteratively throughout the research study, resulting in changes being made to enhance the study design and outcomes.

**Conclusion:**

This paper contributes to the limited knowledge base on embedding PPI into a doctoral research study and within the paediatric setting specifically working in partnership with bereaved parents and carers. Employing an adaptive approach to meet individual PPI needs, building a trusting and respectful partnership, creating shared ownership and investment in the research, are essential components to successful PPI.

**Supplementary Information:**

The online version contains supplementary material available at 10.1186/s40900-024-00620-z.

## Abstract (plain language summary)

Involving patients and the public in research provides the opportunity to develop meaningful outcomes that are relevant to the population being studied. Despite the benefits of patient and public involvement in research, guidelines that support researchers in doing so, lack detail on how to do this effectively. This is particularly important for those new to research such as doctoral students, who have so much to learn in developing a research study. Different approaches and applications to involvement are also likely to be needed depending on the population being studied. There are limited published papers on examples of how doctoral students have engaged and involved patients and the public in the context of their studies, and specifically within the children’s setting, working in partnership with bereaved parents and carers, or those with seriously ill children. This paper offers examples and insights for those new to research in how to involve patients and the public throughout the research cycle. Specifically undertaking research in a sensitive subject of a particular childhood cancer which has poor outcomes and how to incorporate and evaluate successful patient and public involvement in their research activities such as study design and analysis of the results. Parent and carer reflections on their experiences of being involved are also reported and researcher recommendations for approaching and working with a patient and public group are described.

**Supplementary Information:**

The online version contains supplementary material available at 10.1186/s40900-024-00620-z.

## Background

Patient and Public Involvement (PPI) in health research has gained momentum over the last two decades with increased recognition of embedding this within research studies for meaningful and impactful research outcomes. In the United Kingdom (UK) funding bodies expect to see carefully considered plans for integrating PPI throughout the research cycle. Patient and Public Involvement is essential to informing and influencing all aspects of work to deliver impactful research that meets the needs of the public, and patients. In the UK the National Institute for Health and Care Research (NIHR) defines PPI as ‘research being carried out “with” or “by” members of the public rather than “to”, “about” or “for” them [1]. At its core it is a partnership between researchers and the public which includes patients, carers, and members of different communities and organisations depending on the research subject. This partnership approach ensures research is relevant and meaningful to the intended population with the ability to inform and guide how the research is delivered, and as a result, improve research quality [1].

There is a wealth of resources such as frameworks, guidelines, and standards to support clinical and academic researchers incorporating PPI into their studies. For example, the UK standards for public involvement [2] provides a cohesive framework for engaging and involving PPI with suggested ways of working together with continuous re-evaluation of what is working well and where improvements could be made. The NIHR [1, 3] provides a detailed overview of PPI approaches and methods including a list of resources to support writing PPI into research applications, as well as suggestions for reporting and evaluating the impact of PPI. The Patient Experience Library found 536 publications spanning 20 years (2000–2020) of PPI guidance, frameworks, and toolkits for research [4]. Duplications are evident between these with a lack of guidance for those working with ‘seldom heard’ populations where PPI is known to be harder to integrate in research [4]. A systematic review of PPI highlighted that these frameworks are rarely used beyond the authors who develop them, and researchers showed a preference of using multiple evidence-based resources to inform their PPI approach [5]. This suggests researchers may adapt resources and utilise aspects from different frameworks and guidance which are relevant to their PPI population and research activities. These frameworks, guidelines and standards are useful to support doctoral researchers and those new to PPI to think about different ways they can involve people in their research, relating this to their individual contexts to identify how to do this. Despite the wealth of materials available, there is a lack of practical examples regarding the application of PPI in practice which may adversely affect doctoral students and those who are new to involving PPI in research to successfully incorporate PPI activities in their studies. Real-life examples help researchers to think creatively in how to work with PPI members to embed this core component into their research and the resources that are required to do this meaningfully.

To support writing-up PPI work in research, the Guidance for Reporting Involvement of Patients and Public (GRIPP) checklist was developed to provide a structure for reporting and evaluating PPI [6, 7]. This guidance provides a baseline to which researchers can adhere to, with the potential to increase knowledge on ways to integrate PPI within research and across different contexts. Researchers’ who share their approaches to, and applications of PPI, can support doctoral students, and those new to PPI, to develop their skills and understanding regarding this essential component to deliver meaningful research.

### Patient and public involvement in doctoral research

In the UK, involving PPI members in doctoral research throughout the research cycle from study design through to dissemination and implementation is known to improve the credibility of the research conducted [8–10]. There are different approaches to how doctoral students register for a PhD programme in the UK that will impact how, and indeed whether, they engage with PPI. This includes aspects such as funding arrangements, time, knowledge and exposure of the intended research population and resources to involve PPI in their research. Doctoral students may respond to a research call from a funding body or University where the research subject, questions and study design have already been determined. This may mean PPI has been incorporated at the study design stage prior to the doctoral student commencing the research which limits their exposure and experience to this vital research component. Furthermore, doctoral students may not have time allocated to shape and develop PPI within the study, or funding may not have been costed-in, resulting in this key aspect being absent. Alternatively doctoral students may be healthcare professionals who have identified a clinical problem which might be addressed through research. In this case, they are far more likely to have engaged with patients or those with lived experience from the outset. This may involve gathering informal opinions on the problem and whether it is considered important from the perspective of the person with lived experience to investigate further through more formal PPI structures. In some cases, doctoral students might self-fund their doctoral studies and therefore have no dedicated funding for research activities. Patient and public involvement requires funding for doctoral students to effectively engage with PPI activities. Where doctoral students have been funded through national government bodies, for example through NIHR, they will have engaged with PPI from the outset as a requirement of funding being awarded.

Further barriers to embedding PPI in doctoral research include the researchers’ own limited knowledge, skills, and experience in research [11]. In addition to funding and time, other factors which may support or hinder engagement in PPI activities include: supervisor experience of PPI, access to training and the ability to engage with patients or the public to which the research relates [12]. Although funding for PPI is important, having funding does not necessarily mean that PPI will be incorporated effectively. How much PPI doctoral students have seen in practice and been exposed to in other contexts can be important for learning and supporting the translation of this within their own research studies.

The reporting of PPI in doctoral research is rarely published in peer-reviewed journals [8], which may be due to funding and time constraints within PhD programmes, as typically doctoral students (particularly in the UK) present their research within a bound thesis. Alternatively depending on the route the doctoral student takes to register for a PhD and their previous experience, this may limit their PPI engagement and exposure to this essential component within research resulting in few examples being available to draw on. There are few published doctoral PPI examples [8–10, 13] sharing different aspects and approaches to PPI from which doctoral students can gain insight and knowledge. How doctoral students approach and engage PPI will be dependent on the type of study they are conducting and the subject area. A lack of examples of PPI within doctoral studies across the range of study methodologies and subject areas, is a potential barrier and may deter those who want to engage and involve PPI but struggle to know how. Sharing effective examples and offering guidance on how to incorporate meaningful PPI, could help those new to conducting their own research to build confidence and knowledge to involve PPI contributors. Given the importance and arguably moral imperative of PPI throughout the research cycle, highlighting approaches and real-world applications is essential to equip doctoral students to think more creatively about ways to involve PPI in their own work. Engaging PPI from the outset of a researchers’ career may also enhance their ability to successfully implement PPI in future research work.

### Patient and public involvement in different contexts

The challenges and approaches to PPI are likely to differ depending on a given research study’s target population. Sharing examples of PPI across different healthcare settings is important, particularly as the majority of published PPI literature is within adult settings [8, 9, 11, 14]. These examples include PPI contributors who were overweight to design a dietary intervention [8], family carers of someone with cancer [8], Black, Asian and Minority Ethnic groups to focus on inclusion and diversity in research [9], had a rheumatic condition [10], experience of administering medications with a focus on improving safe use of medications [11] and those with adult cerebral palsy [13]. The majority of these examples took a reflective approach from the researcher and PPI contributors [9–11, 13] with one informed by the concepts defined by Hughes and Duffy [15]. Whilst all of these examples were published after the GRIPP checklist was developed [6, 7] only one reports using this [8] which suggests a lack of awareness of these reporting guidelines. Consensus of successful PPI across these examples included planning PPI aims, meetings and activities from the outset [8, 9, 11, 13], ensuring resources such as finances were available to support PPI engagement [8, 10] and maintaining a record of PPI activities and outcomes to monitor impact [8, 9]. While these examples provide meaningful ways to engage and involve PPI, one of the key differences within the paediatric setting is that of parents as proxy decision-makers for their child. Their burden of responsibility is different from people who are making decisions on their own health issues or experiences. Research studies within paediatrics can be complex due to the potential burden of participation versus expectations alongside care demands placed on parents and carers whose child is unwell [16]. These complexities may contribute to higher withdrawal rates or decreased participation in research studies within the paediatric setting [16]. The incorporation of PPI from the outset of study design could influence and address issues such as recruitment and retention to increase research participation within the paediatric setting.

Within the paediatric cancer landscape, partnerships between parents and patients—those with lived-experience—and researchers and/or research institutions have long been established. Parents often raise money to fund research [17] and may stipulate how they would like this to be used, for example within a specific diagnosis, or particular aspect of treatment. This is seen particularly with parents and families whose child has died from the disease as a way of keeping their memory alive and advancing treatments for future children. An example of this is parents of children diagnosed with high-risk neuroblastoma, an extra-cranial solid tumour most commonly arising from the adrenal gland with metastatic disease at diagnosis [18]. Survival rate is 50% [19] despite an 18-month intensive multimodal treatment protocol. In children where the disease relapses or is refractory (does not respond to initial treatment), the survival rate decreases to around 10% [19]. Bereaved parents in this context raise charitable funds for research or contribute in other ways such as through research PPI groups or working with charities. While funding research is not PPI, having no active ongoing influence or involvement in research design and delivery, it demonstrates that there is broad investment in advancing the research landscape within children’s cancer among the parent community. There are UK children’s cancer charities who fund research which stipulate the need for thoroughly considered and integrated PPI to be embedded into research funding applications alongside national bodies such as NIHR. These charities have PPI established groups who review the plain English summary and PPI sections of research applications. This highlights the emphasis placed on PPI within this speciality in addressing research questions which are relevant and meaningful to the population being studied recognising the need for thoroughly considered PPI to be embedded across research studies.

### Aim of this publication

This paper aims to contribute to understanding of how PPI can be effectively incorporated by doctoral students into their research studies. It extends the current evidence base of published examples on PPI in doctoral research [8, 9, 11, 13] providing an example of PPI throughout the research cycle and recommendations for doctoral students to approach and develop their own PPI group. It illustrates how it is possible ‘to do’ PPI meaningfully even in contexts where there are significant challenges, such as in paediatrics where barriers such as expectations and motivations as a parent caregiver and burden of participation differ to adult settings [16].

Our paper describes the use of PPI within a paediatric focused doctoral research study, reporting and evaluating involvement throughout the research cycle. Personal reflections from PPI contributors on their perspectives of involvement are included. Experiences documented within the paper may help doctoral students engage PPI in a variety of ways to create a partnership approach with genuine impact on meaningful research outcomes.

## Overview of the research study

This research was funded by NIHR as part of a personal fellowship awarded to HP. The focus of this doctoral research was on parental treatment-related decision-making in respect of a child with relapsed or refractory neuroblastoma, a poor-prognosis cancer. Typically, children living with this diease are under five years of age and therefore not actively involved in the decision-making process. For relapsed and refractory disease there is no standard treatment pathway, but various options are available such as clinical trials and experimental therapies. Parents become involved in making treatment decisions for their child due to having no standard treatment with no clear endpoint. These decisions are often repeated depending on treatment availabilities, response to treatment, and aggressiveness of their child’s disease.

The study had two aims: (1) To identify, describe, explore, and explain how parents made treatment decisions when their child had relapsed or refractory neuroblastoma (Phase one); (2) Develop an intervention prototype for parents to support their treatment decision-making and conversations with healthcare professionals in the context of treatment choices (Phase two). Phase one included a literature review and qualitative semi-structured interviews with parents making treatment decisions. Phase two followed the Medical Research Council (MRC) Framework [20] for developing complex interventions co-designed with a parent stakeholder group.

Ethical approval was received by University of Southampton (study sponsor) and NHS Health Research Authority London Bloomsbury Research Ethics Committee (19/LO/1715).

## Aims of patient and public involvement within this study

The aims of PPI in this study were initially devised by the researcher based on their clinical experience of this being a sensitive subject to research and through reading NIHR documents on PPI [1–3]. These aims were later shared with the core PPI group for group consensus which identified and supported ways in which PPI was embedded throughout the study.

- To obtain parent feedback on whether the research subject was appropriate and considerations for the study design.
- To integrate PPI within each stage of the research cycle for consistent meaningful impact being mindful of the sensitivity of the research subject.
- To evaluate the impact of PPI within this study and share our approaches with a wider audience.
- To identify ongoing PPI requirements for successful implementation and dissemination of the research findings and intervention developed.

## Methods

**Fig. 1:**
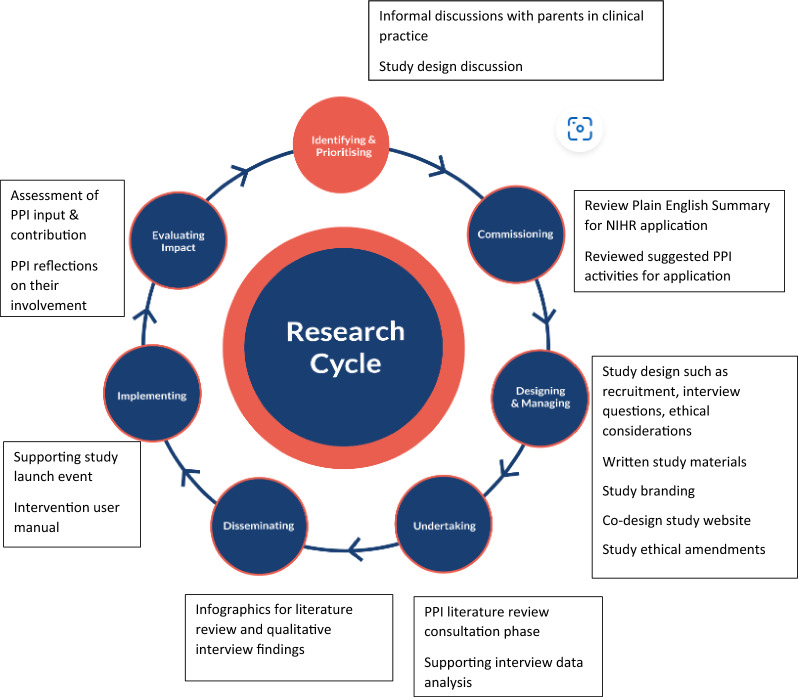
Research cycle for REDMAPP study

**Fig. 2:**
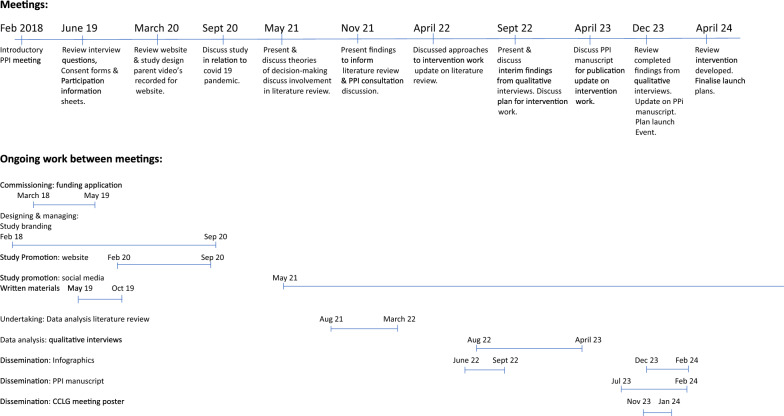
Timeline of PPI involvement in the study

Patient and Public Involvement was informed by the research cycle [1]. This consists of seven stages: (1) identifying and prioritising; (2) commissioning; (3) designing and managing; (4) undertaking; (5) disseminating; (6) implementing; (7) evaluating impact. Examples of activities PPI members engaged with in the research cycle can be seen in Fig. 1. Many stages were iterative as the research study evolved. For example, the iterative nature of qualitative data analysis meant PPI members were involved in this work over nine months with regular meetings to review and discuss codes, data and themes as the analysis developed. These meetings impacted how the analysis developed. Evaluation of the approach to PPI was captured through audio recordings from the meetings with written notes which were reflected on with the group to support the writing of this publication. A PPI timeline can be found in Fig. 2 which shows the PPI meeting topics and the work which happened between meetings. Patient and Public Involvement was reported using the GRIPP2 short form checklist [7] (Additional file 1).

The NIHR briefing notes for researchers [1] suggest ways of involving PPI within each aspect of the research cycle. This provided a starting point for ideas which were adapted by the researcher (HP) based on their clinical experience of working with this parent population. For example, informal discussions with parents in clinical practice were conducted to find out whether they found decision-making difficult, and to establish whether research in this area was important and could be of real benefit. These informal discussions led to the concept of the research study being identified as parental treatment decision-making. It was important to gauge whether this was an area to address through research before convening a group of parents to explore such a sensitive subject in more detail.

### Incorporating and involving PPI from the outset

Engaging parents from the outset felt important given the sensitive nature of the research. The purpose was to identify, based on their experiences, whether this was a subject that required addressing through research [1]. The concept of the research study (parental treatment decision-making) was presented at the Neuroblastoma Parent Education Conference organised by the charity Solving Kids’ Cancer UK, in November 2017. This included a call for PPI to help develop and inform the study at an introductory face-to-face meeting scheduled for February 2018. Criteria for PPI were parents or carers who had experience of making treatment decisions for their child diagnosed with neuroblastoma. Four months between the call for involvement and the meeting taking place allowed time for parents and carers to consider their involvement and organise personal schedules to attend. Additional calls made through social media posts, were shared by charities and parents to broaden reach and engagement. In total 13 parents and carers responded with eight having attended the conference.

### Identifying and prioritising: introductory PPI meeting

The aim of the introductory meeting, attended by nine parents and carers, was to obtain feedback on whether the research subject was appropriate and to gather initial design considerations. This two-hour meeting was held in London on a Saturday morning, in February 2018, with part funding from the Research Support Service [21] Enabling Involvement Fund. Additional funding came from the researcher’s NHS Trust where there were funds allocated to the development of this study donated by a family. The meeting was audio-recorded (with consent) to enable the researcher to ensure all topics raised by parents were considered and nothing was missed. The meeting concluded with a presentation of the research cycle [1] showing ways in which PPI could be embedded throughout the study. The group were asked to consider their ongoing involvement in the study, with five parents/carers going on to form the core PPI group.

Given the sensitivity of the research subject, a clinical nurse (EP) working with the researcher attended the initial meeting to provide support to attendees if required. This role was introduced at the meeting outset highlighting if parents needed time out from the meeting EP was there to accompany them to provide a listening ear, talk through their feelings and emotions should this be required. The transferable nursing skills of active listening, being non-judgemental, treating people as unique individuals and clinical experience of working with this parent population meant this role could support parents with compassion. This role evolved such that the clinical nurse attended all subsequent meetings, playing a key part in the PPI approach through listening to the group interactions and sense checking discussions providing valuable support to the whole group..

### Establishing a core PPI group

The core PPI group consisted of four parents (two mothers, two fathers) and one grandparent all of whom were bereaved between 6 months and 10 years at the outset of the first core study meeting in June 2019. There is no definitive number of PPI members considered to be ideal when undertaking research. The NIHR briefing notes for researchers [1] suggests involving more than one person in order for people to have choice in their involvement within the different stages of the research. The number of PPI members involved is likely to depend on factors such as the research subject, size of the population affected, and methods used to promote PPI. All group members had broad experience of making repeated treatment decisions in relapsed or refractory neuroblastoma. They were geographically spread across the UK which increased diversity through having experience of multiple children’s cancer treatment centres. Previous research has shown fathers’ voices are often seldom heard in this type of research [22–25], therefore it was important to have them represented within the group.

Throughout the study, approximately six monthly in-person meetings were held with the first meeting in June 2019. Meetings were in-person until March 2020. The COVID-19 pandemic necessitated a transition to virtual meetings, which continued for the remainder of the study. All meetings were 2-h held on a Saturday morning, audio-recorded, with consent from the group, so they could be reviewed afterwards by the researcher, building on experience from the initial PPI meeting. Meetings were structured with an agenda devised by the researcher and shared with the group three weeks prior to the meeting. Each meeting commenced with a study update provided by the researcher with opportunity for PPI members to ask questions and troubleshoot specific areas such as recruitment. The focus of each meeting can be seen in the PPI timeline (Fig. 2) which corresponded with current study work, work which remained ongoing or was yet to be completed. Work with PPI members also took place outside of main meetings through email, virtual, and in-person meetings, depending on the precise nature of the work and input required. Group members’ time and expenses (travel and subsistence) were paid in accordance with the NIHR suggested payment rates [26].

### Ethical considerations

The complexity of including bereaved PPI members has been seen in other studies [27, 28]. All PPI members were bereaved and to maximise their involvement individual needs were considered. Meetings were planned in advance to avoid and work around significant dates for example birthdays, anniversaries, and holidays such as Christmas. Agendas and other documents were provided in advance of a meeting for the group to review. Providing adequate time was crucially important so group members were not overburdened and did not feel pressured to review materials that were potentially hard to read and process in a short timeframe. When work took place outside of the main meetings, these meetings were organised based on PPI member preference to minimise distress when engaging with aspects of the study that could evoke emotions.

The emotional investment of PPI members was continually addressed during meetings creating a safe space to share their personal stories, talk about their children and express the raw emotions they experienced and continue to experience through bereavement. The researcher and clinical nurse had extensive experience of communicating with parents and carers at all stages of a child’s diagnosis, treatment and following a child’s death from their clinical practice. The skills around the sensitivities of these communications were transferable skills employed implicitly whilst working with the PPI group.

## Results and impact of PPI involvement

A Total of 11 PPI meetings were held between February 2018 and April 2024. Nine meetings were attended by all PPI members and three meetings a member was unable to attend last minute due to personal circumstances. In such circumstances, the researcher either met 1:1 to discuss the outcomes from the meeting or provided an email summary of the meeting with opportunity for group members to feedback on any topics which were discussed.

This section details the PPI activities relating to the research cycle: (1) commissioning of the research funding application; (2) designing and managing of study materials, branding, promotion, and recruitment; (3) undertaking with involvement in the literature review and qualitative data analysis; (4) dissemination and implementation of the study findings; (5) evaluating impact through PPI group reflections which led to devising PPI recommendations for doctoral research within a sensitive subject. Contributions from PPI members included co-design, reviewing materials to provide input and suggestions to develop and enhance the study. These contributions influenced and informed the study direction and outcomes as described below. Table 1 summarises the PPI activities relating to the research cycle detailing who was involved, the impact and evaluation in the study.

**Table 1:** Summary of impact and evaluation of PPI activities

PPI activities	Approach	Impact	Evaluation
Identifying the research problem	Informal discussions with parents in clinical practice	Highlighted a need to support parents in making treatment decisions for their child	Concept of research defined: parental treatment decision-making Further validation of this research topic and potential impact to discuss at formal PPI meeting
Identifying and prioritising: Study design	Introductory PPI meeting PPI group meetings	Approach parents each time they make a treatment decision to reduce burden of participating in a longitudinal study Provide flexibility in when/where interviews take place Recruit parents via their child’s cancer treatment centre	All suggestions incorporated into the research protocol which received HRA/REC ethical approval
Commissioning: Funding application	2 PPI group members reviewed via email	Amendments to the Plain English Summary language, shorter sentences, and structure to increase accessibility	NIHR funding approved
	PPI group meeting	Reviewed and agreed suggested PPI activities within the study	NIHR interview panel commented on how thoroughly PPI was considered within the study
Designing and managing: Study branding	PPI group meeting, group email discussions	Study acronym and logo devised for study identity and PPI group ownership	REDMAPP acronym and logo created for use across social media platforms and all study related materials
	1 PPI group member via email	Flow diagram to visualise pathway of how parents make treatment decisions to support need for the study	Flow diagram used in researchers’ NIHR funding application, presentations to study sites
Designing and managing: Study promotion	PPI group meeting, 1:1 meetings with researcher and parent, group email discussions	Content written for the research study website by parents for parents to support accessibility Social media written content reviewed to improve language for readability and accessibility	Study website www.redmappstudy.co.uk
	2 parents with videographer company	Parent videos talking about their decision-making experience and involvement in the study to support parent awareness and engagement of the study	Videos available on the study website and used in social media posts
Designing and managing: Recruitment	PPI group meeting	Ethics amendment for parents to self-refer to the study	No parents self-referred to the study suggesting importance of human contact to explain participating in a sensitive topic
Designing and managing: Written materials	PPI group meeting	Parent information sheet simplified to improve readability	Six parents in the study commented how easily the Parent Information Sheet was to read
Designing and managing: Interview questions	PPI group meeting	Wording changed to be neutral and non-judgemental List of prompts devised if needed in interviews	Eighteen parents interviewed for the study with ten parents commenting how positive being interviewed had been on their psychological well-being
Undertaking: Data analysis (literature review)	1:1 parent and researcher multiple virtual and 2 in-person meetings	Reviewed data from literature review and co-developed findings offering suggestions to theme names	Strengthened findings having parent involved to be relatable and relevant to parent population
	PPI group meeting	PPI consultation phase for literature review highlighted lack of knowledge on if and how emotion informs or influences parent decision-making Additional research question added to parent interviews	Gap in knowledge addressed within the study which was important to parents
Undertaking: Data analysis (parent interviews)	2 parents multiple virtual meetings	Reviewed coded data for interpretation to discuss researcher’s thinking and approach to analysis Discussed components of themes and grouping of codes to provide a narrative of parent treatment decision-making	Supported researcher’s critical thinking and approach to analysis
	PPI group meeting	Reviewed findings from parent interviews identifying theme names which could be subjective and cause distress to parents	Theme names reviewed and jointly agreed between parents and researcher
Dissemination and implementation: Literature review and parent interviews	Parents and researcher virtual meetings (those involved with data analysis)	Suggested visuals to illustrate findings from literature review and parent interviews	Illustrations incoporated these visuals and supported disseimination within the parent community
Evaluating Impact	PPI group meeting and group email discussions	Offering reflections on PPI involvement within the study	Reflections incoporated within this paper and PPI recommendations in doctoral research within a sensitive subject were devised

### Identifying and prioritising: introductory PPI meeting

Discussion on the study design considered barriers and enablers to participation, interview questions and location, approach to recruitment and ways to minimise burden of participation. Additional file 2 details feedback on these aspects and how they were incorporated into the study design.

### Commissioning: funding application

Two group members (CB, CK) reviewed the Plain English Summary for the researcher’s NIHR doctoral fellowship application. Based on their suggestions, amendments were made to language and structure to increase the accessibility of the summary. For example, ‘standard-of-care treatment’ was changed to ‘initial treatment’ and shorter sentences used to make information easier to read. At the application stage it was difficult to foresee the extent to which PPI would be embedded into the study as the research progressed. To ensure appropriate funding was available to support future PPI activities, 10 days of PPI time per year was costed into the application, in addition to payment for meetings, travel expenses, and subsistence. The group reviewed the suggested PPI activities included in the fellowship application to ensure these were feasible and meaningful. For example, six monthly meetings, commitment to input and review study materials, co-design of the study website, input with data analysis and dissemination strategies. Reviewing these activities in partnership ensured that PPI members knew what to expect, what their input would be, and how it might evolve as the study developed. It was also important for PPI members to know that their involvement would be valued and remunerated accordingly.

### Designing and managing

Study design and management were iteratively discussed at each PPI meeting, with opportunities for input and recommendations. The researcher provided a study update and discussed ongoing developments on areas that were problematic, for example, slow recruitment during phase one. These iterative discussions changed and enhanced the study design across several aspects, examples of which are highlighted below.

#### Study branding

**Fig. 3:**
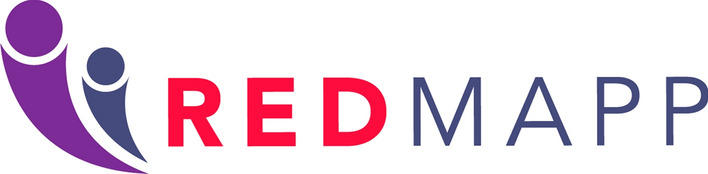
REDMAPP logo

**Fig. 4:**
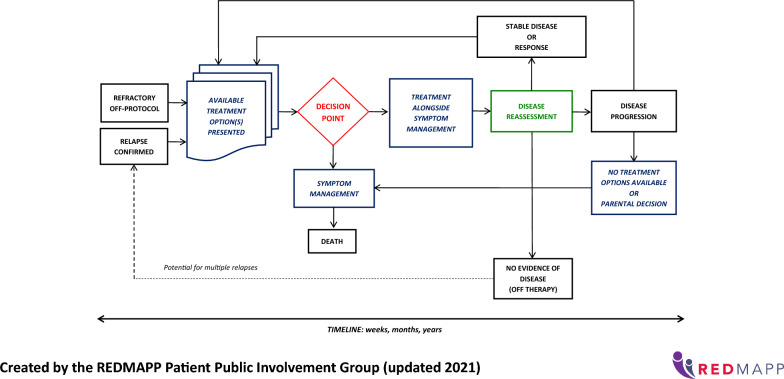
Flow diagram of parent treatment decision making

At the outset of the study, the group devised the acronym, REDMAPP to give the study its own identity. This was supported with a logo (Fig. 3) developed by a graphic designer who produced eight options for the group to choose from based on their guidance of what the logo should represent. This fostered an early sense of co-ownership and helped the researcher to build a partnership approach. One parent (LK) developed a flow diagram to visualise how parents are involved in making repeated treatment decisions (Fig. 4), which was reviewed by the whole group. The flow diagram was included in the NIHR application and various study materials to aid understanding of the research subject.

#### Study promotion

The group co-designed the study website: www.redmappstudy.co.uk which aimed to provide information about the study to the wider parent community to support engagement and recruitment. Health literacy was an important consideration to ensure as many parents as possible were reached. Two group members (LK and KC) were videoed talking about their decision-making experiences and reasons for being involved in the PPI group. Videos were uploaded onto YouTube: https://youtu.be/HJIzIZUnMNQ [29] and https://youtu.be/AkTcLHUarlA [30] and hosted on the study website.

As the study progressed the group felt it would be good to have a social media presence (Twitter: @redmapp_study; Facebook: REDMAPP Research Study; Instagram: @redmapp_study) as parents often use these platforms to engage with other parents and share experiences. The purpose was to increase awareness, disseminate findings, and request involvement in specific aspects of the study, for example, user testing for the intervention work. Having a social media presence resulted in 171 parents following the pages internationally via Instagram and Facebook and 12 additional parents responding to requests for involvement.

#### Recruitment

Discussions on participant recruitment within the PPI group highlighted the need for flexibility to allow parents to participate at any point in their treatment decision-making pathway and not only at the first treatment decision. This was built into the study design with support from the PPI group. This design considerations resulted in eight parents participating after their first treatment decision, and three parents being interviewed twice. When non-essential research studies were paused during the COVID-19 pandemic, the PPI group suggested submitting an ethics amendment which would enable parents to self-refer to the study. This was acted on and approved. Despite this amendment no parents self-referred to the study suggesting the importance of someone explaining the study to them as opposed to using social media for calls to participate.

#### Written materials

Parent information sheets were reviewed by PPI members and simplified to help improve readability. Six parents who participated in the study commented that this helped them to navigate the information more quickly and easily.

#### Interview questions

Interview questions were drafted by the researcher based on clinical experience and evidence from the literature. These were subsequently reviewed by the PPI group. They provided critical feedback regarding the use of the word ‘why’ in relation to questions on how parents made their decisions, which could be construed as judgemental with potential for parents to feel criticised for the decisions they had made. For example, questions such as ‘*why* did you decide on that treatment over the other options available’ and ‘*why* was that important to you when making a decision’ were changed to ‘talk me through the latest treatment decision you have made’ and ‘what was important to you when making that decision’. The group also devised a list of prompts based on personal experience that could be used during the interview, if required.

### Undertaking the research: data analysis

While undertaking the literature review it became apparent that the nature of parent decision-making has changed over time. For example, compared to thirty years ago there is now greater emphasis on shared decision-making between parents and clinicians [31, 32]. As a result, the researcher asked the group for their involvement to ensure findings were relatable to decision-making in today’s context. Through a combination of virtual and in-person meetings, a group member (CK) reviewed and interpretated the extracted data in the literature review and co-constructed findings with the researcher. This work was published [33] with CK as a named co-author.

The review findings were discussed with the wider PPI group who highlighted that the role of emotion in parent treatment decision-making was absent from the literature. They felt this was important to acknowledge and address given the emotional impact of having a child with cancer. As a result, an additional research question was added to the parent interview guide which addressed: ‘explore, explain and describe the role of emotion in parent treatment decision-making when your child has relapsed or refractory neuroblastoma’.

Given their valuable input to the literature review, the PPI group was asked to participate in the data analysis from parent interviews. The purpose was to support the researchers’ critical thinking on the interpretation of the coded data and themes which developed from the analysis. Two PPI members (LK and KC) agreed and took part in a series of regular virtual meetings with HP to review and discuss codes and their associated data. This helped the researcher to see patterns between codes resulting in refining and re-grouping codes to provide a cohesive narrative across the dataset.

In a later phase of analysis, the PPI group contributed to the identification of theme names bringing awareness of the sensitive nature of the research subject and how certain words or phrases might be interpreted by parents. For example, ‘empowerment in decision-making’ and ‘advocating for their child’ were seen as subjective and carried the potential to induce guilt or suffering if a parent’s interpretation was that they should or could have done more for their child. Alternative suggestions included involvement and responsibility in decision-making with clear interpretation within the analysis of what these concepts meant in relation to the study findings.

### Dissemination and implementation

Disseminating study findings amongst the parent community were discussed by the group. Drawing on their own experience they acknowledged that parents were unlikely to read large paragraphs of text and to actively engage them would require thinking more creatively in a visual format. Infographics were used to share study findings, co-created by PPI members (CK, KC, LK), the researcher, and a professional illustrator. The infographics required careful consideration of how to represent the findings in a way that would neither offend nor inadvertently cause emotional distress to parents.  Illustrations were made of the literature review findings https://youtu.be/H27rR3ytsTs?si=CqCi8EKi3kbmzbod [34] and parent interviews https://youtu.be/kf1Tdhbfnqo?si=wrLWAjIenqGvxMT3 [35] which received overwhelmingly positive feedback from parents who commented that they were able to relate and understand the findings clearly without detailed explanation. Findings were also shared with Solving Kids’ Cancer UK Parent Involvement Forum to further support dissemination within the parent community.

To share findings with a wider audience, an official launch event of the study findings is planned inviting parents, healthcare professionals, charities, researchers, and other relevant stakeholders. The launch will be co-hosted with PPI members who will share experiences and the impact for them of being involved in the study. Such an event is a way to shine a light on the importance of incorporating effective PPI in doctoral research work.

**Fig. 5:**
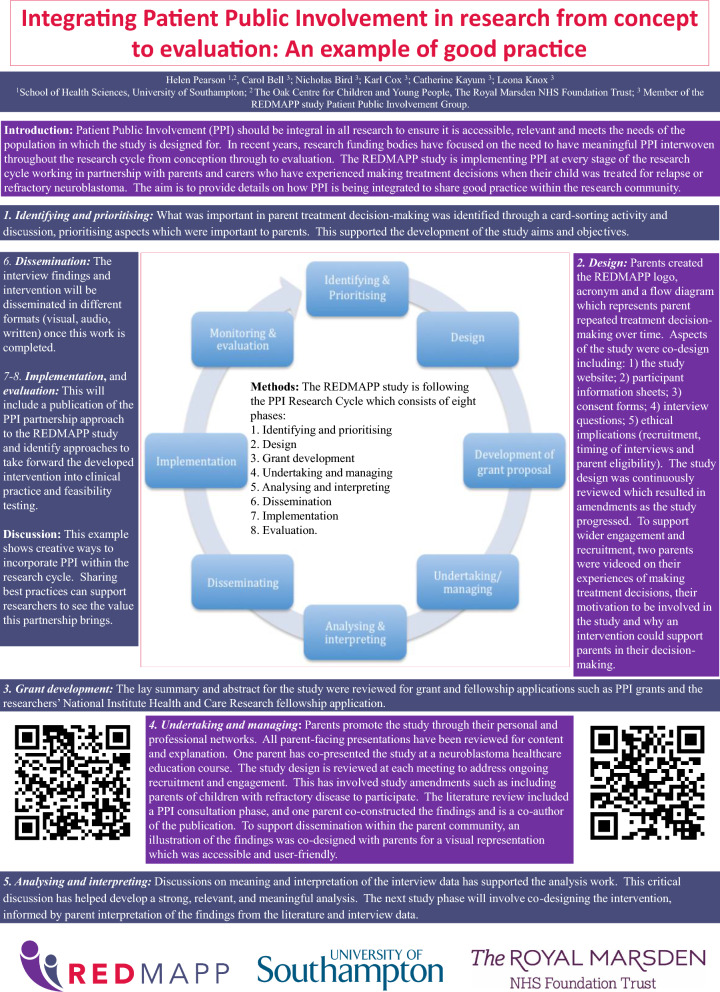
CCLG PPI poster

The approach to integration of PPI taken throughout the research cycle was presented to clinical and academic researchers working in paediatric oncology in a poster entitled ‘Integrating Patient Public Involvement in research from concept to evaluation: an example of good practice’ at the Children’s Cancer Leukaemia Group (CCLG) Annual Meeting 2023 (Fig. 5). This led to discussions on ways to engage PPI from the outset of developing a study, including how to factor in appropriate funding.

## Future PPI work

Ongoing and future PPI work is integral to translate this research into clinical practice. This work requires facilitating use of the intervention website within the parent community and raising awareness with educating healthcare professionals and charities about the website to advocate for its use with parents. An intervention manual combining visual, audio, and written formats to support parents and healthcare professionals in engaging with the website will be developed.

The MRC framework for development complex interventions has four phases [14]. The first phase has been achieved within this doctoral research study, developing the intervention. The three remaining phases: feasibility, implementation, and evaluation will be addressed post-doctoral. Further research is required to enhance and refine the intervention to fully integrate this into clinical practice. The PPI group will support the  study design of the next phase of this work.

## Evaluating Impact: PPI group reflections

In the April 2023 PPI meeting, plans for writing this PPI manuscript were discussed. This involved drawing out the methods of how PPI was incorporated within each stage of the research cycle and evaluating its impact. Group members were asked to think about and share their reflections of being involved in the study via email to the researcher. This was done individually so PPI members could reflect on their involvement without being influenced by other members of the group. Table 2 provides reflections from group members. The reflections were captured in an unscripted way which the researcher organised into common themes informing a synthesis of the group reflections.

**Table 2:** Reflections from PPI members

Reflections	
Personal experiences	“Being engaged in the REDMAPP study holds immense significance for me. I know what a terrifying experience it is to make what felt like life-or-death decisions for my child, and the researcher’s commitment to transforming this experience for future parents resonated deeply with me.” “I was very keen to be involved because I was, and still am passionate about wanting to help parents & families navigate the difficult treatment journey for their child. As a grandparent of a child with relapsed neuroblastoma, it fell to me to research treatment options while the parents focussed on parental care, hospital visits etc. It had such an impact on me, I was very determined to make a difference to the journeys of families in the future.”
Sense of purpose	“Contributing allowed me to shape and inform the work based on my own experiences, ensuring my turmoil was not in vain and that it could potentially ease the journey for others. Being part of a dedicated team comprised of professionals and individuals with lived experience, all united in pursuing the same goal, was a powerful and rewarding experience.” “I wanted to be involved to improve things for future parents and I have a passion for involving patients in research and making information accessible to a wide range of patients. My involvement in this study has strengthened that.” “I’ll be eternally grateful for the opportunity to participate in this work. I felt that the contribution of all parents was valued & there was a strong sense of unity, common purpose, passion & commitment to doing the very best we could to support the study.”
Feeling listened and heard	“The researcher placed a very tangible value on our participation, by ensuring we were well informed and heavily involved in all stages, our ideas were heard and sometimes changed the course of the study.” “I appreciated the open forums for discussions, as I have a real dislike for PPI being reduced to rating scales and questionnaires! I like that our group observation through the literature review on previous research being sanitised and lacking in the emotional dimension led to valuing that information in this study.” “All members contributed through listening and reflecting on what each other said. We understood that the research was not about our own experiences directly, but that our role was to bring intrinsic knowledge and understanding to help shape the project in terms of design, participation, interpretation, and dissemination.”
Enabling a flexible approach	“I particularly valued the researcher’s approach to creating a safe environment where everyone could freely discuss a subject that has an enormous personal emotional burden. This added to the authenticity of the study and avoided limiting its scope. Importantly, the flexibility provided, including breaks when needed and tasks that were designed to be manageable, demonstrated a genuine understanding of our needs.” “I appreciated the sensitivity and flexibility that allowed me to stay involved, in terms of taking enough time, working around more difficult times.”
Collaborative partnership	“I enjoyed the sense of team we developed and the chance to draw on the different strengths within the group.”

## Clinical nurse reflections

To safeguard parents and carers involved in research of a sensitive nature, the researcher engaged a clinical nurse (EP) to support PPI members from an emotional and psychological perspective. This involved accompanying parents out of meetings if they became distressed or contacting parents through direct message in virtual meetings or via email after the meeting. Identifying a person for this role was important to ensure parents could be cared for enabling the researcher to continue with meetings where appropriate. Although the purpose of this role was to support parents emotionally and psychologically this was not required in any of the meetings. Over time this role evolved to become an integral and valued member of the group, contributing more broadly to reinforce the partnership approach. They provided clarification as topics were discussed and bought new insights from their  own perspective and professional experience. Table 3 captures the clinical nurse’s reflections of involvement within the group.

**Table 3:** Reflections from clinical nurse

Reflections	
Perceptions of PPI	“When working with patients in clinical practice you are always trying to ‘fix’ it, make it better but within PPI all we can do (or so it seems) is listen. Learning to listen without excuses, without trying to fix and most importantly without judgement. As a healthcare professional, instigating PPI is in some ways like taking a leap into the unknown. You must be prepared to leave your preconceptions at the door and come to the table truly willing to hear what the participants are wanting to share. Not knowing what was going to be brought up by the participants felt scary at first. Our confidence grew over time, as did the confidence of the PPI members creating one working team with all participants mutually supporting and challenging each other seeking the best outcome.”
Collaborative partnership	“The group came together as one team, developing an open, honest, and trusting partnership. I valued the sense of shared purpose as we all worked together to try and find solutions to ease what can be a very difficult time for families and healthcare professionals alike.”

Parents and carers viewed the involvement of a clinical nurse as a positive, changing the group dynamics: “The clinical nurse role helped to bring out more from the group, helping to probe. Her personal attributes and ability to take on that supporting role alongside the researcher made a real difference in terms of it not just being a single healthcare professional as the researcher and a group of parents. I think it changed the dynamic in that we weren’t always hearing from the researcher when we weren’t speaking ourselves.”

## Researcher reflections

As an experienced clinical nurse practitioner who has engaged with parents for many years in that setting, this was the first time engaging with parents in the context of PPI for the researchers’ doctoral study. Conducting qualitative research in the field of children’s cancer can be highly complex and an emotive subjective involving a diverse population of parents which required careful thought in the study design. Working with a PPI group felt a natural part of the research process to ensure a sensitive approach and what began as involvement following PPI guidance became a partnership in all areas of the research study, creating a trusted, valued, and earnest team dynamic. Having an open mind, being prepared to listen and adapt the research study based on PPI suggestions influenced the research in a meaningful way. The researcher was new to developing and managing a PPI group, having a trusted colleague helped with group observation, note-taking, and identifying topics that were mentioned by group members but not discussed thoroughly. It also provided an opportunity for the researcher to debrief and discuss approaches to ongoing engagement and partnership working within the group.

Working with bereaved parents and carers required additional time for reflection, recognising their emotions particularly when sharing the findings from the qualitative interviews. Taking extra breaks when needed during meetings and organising meetings in advance on a date that all the group members agreed on helped to support parents and carers to participate in the group and feel valued for the contributions they made.

Prior to undertaking this research, the researcher had exposure of active PPI through interactions with charities, the neuroblastoma parent community and wider involvement in the UK neuroblastoma research community. This exposure meant the researcher was able to utilise existing relationships to support the development of the PPI group for example presenting the research idea at the Neuroblastoma Parent Education Conference. Building relationships across organisations and seeing PPI in action in other contexts supported the researcher’s understanding of ways to approach PPI at the outset of the study to develop a group. This experience was important to facilitate working with bereaved parents within a doctoral study where the researcher was new to research and undertaking qualitative research on a sensitive topic involving a specific small vulnerable population of parents and carers whose child’s survival was poor. The contributions of PPI members were acknowledged in all journal publications, including co-authorship where appropriate. This was important as this study was a partnership approach between the researcher and parents and carers who invested significant time, emotion, and energy through their involvement.

## Synthesis of PPI reflections

Reflections from the PPI group highlighted why parents and carers became involved in the study, they wanted to help future parent and families and were invested in what the research study aimed to achieve. Having affiliation with the research topic is important for PPI members to be able to relate to and see the importance of the research being conducted. In doing so PPI members are more likely to contribute and continue with their involvement as opposed to those who have no affiliation with the research topic. Group members spoke to their desire to help future parents and carers through their own experiences which has not been alluded to in other PPI doctoral publications. This might be attributed to the specific context of this PPI, bereaved parents who understand the complex and difficult decisions they had to make for their own child and wanting this to be better for others in the future.

The group also spoke of having meaningful impact and the opportunity to contribute to activities in a way that added genuine value. Being listened to and heard, working flexibly, the researchers’ compassionate approach to the research topic and PPI group supported building a collaborative partnership. The nursing skills of the researcher and clinical nurse through their understanding of different levels of knowledge, adapting language, being an active listener, facilitating time for all voices to be heard and extensive clinical experience working with this parent population nurtured this adaptive approach to PPI. Adding genuine value through contributions can be seen as having a sense of purpose within the research study which has been seen in other doctoral studies [8, 9].

From these reflections, common themes were devised to inform the recommendations for PPI in doctoral research within a sensitive topic. These recommendations were developed by the researcher and reviewed by the group for consensus.

## Discussion

Published papers reporting the incorporation of PPI into doctoral health and social care studies are predominately within the adult setting [8, 9, 11, 13], which has limited application for paediatric research [16]. This paper illustrates how PPI was approached and applied within a paediatric focused doctoral research study throughout the research cycle providing strong examples of PPI integration and the impact this can have when there are adequate resources available. Working in partnership with bereaved parents and carers requires time, compassion and the creation of a flexible approach that facilitates different ways to be involved. Patient and Public Involvement activities have been reported as per the GRIPP2 reporting guidelines [7] to provide a consistent approach in reporting the integration of PPI within research. Personal accounts from PPI group members show the importance of working in partnership with people who have lived experience of a research subject, aligning with guidance on PPI [1, 2]. These reflections also show that bereaved parents and carers want the opportunity to be involved in research after their child has died, to help future parents and utilise their experience in a positive way.

Meaningful PPI relies on building and developing trust and respect between PPI contributors and researchers [36] recognising the contributions PPI members make to research. The reflections from PPI members spoke of the importance of being listened to and heard which created a collaborative partnership of mutual trust and respect. To truly involve PPI meaningfully took time to nurture and develop a partnership with the group having a shared vision and sense of purpose. Time also needed to be factored into the study timeline, forward planning and preparing PPI members so they had time to think and reflect to enable meaningful discussions and input to develop new ideas and approaches which continually improved and enhanced the study.

Impact started with validating the research study conceptually looking at the potential impact this could have for the parent population. The importance was to ascertain whether parents who have been through treatment decision-making in this context believed in the study and what it could deliver in terms of helping and supporting future parents and families making these decisions. This was fundamental for successful PPI within this research study and indeed should be in any research. Active PPI comes through genuine investment in what the research is seeking to achieve, in this study helping parents and their child affected by relapsed or refractory neuroblastoma. Impact ultimately in whether PPI activities have helped shape and deliver the research aims is yet to be determined based on ongoing evaluation of the study outputs, in particular the website intervention to support parents and carers who are making these decisions. The lifecycle purpose of PPI is about why and if the research should be conducted with a focus on the assessment of public and societal value the research brings which extends beyond funding and the academic necessity of incorporating PPI to satisfy a tick box.

This doctoral study was conducted within a small patient and parent community, and some members of the group knew each other prior to working together in this capacity. Relationships strengthened over the course of the study through regular communication via meetings, small working groups, and emails. Group dynamics and relationships were also vital for a cohesive PPI partnership [9]. The role of the researcher was to ask, probe, listen and learn. Those involved in PPI need to be listened to and know they are being heard. Showing that their opinions and views are valued, and they are an integral part of the study team, has to be demonstrated by actions not merely words. Incorporating PPI as a ‘tick box’ exercise or a means of appeasing funder’s requirements through a tokenistic approach will inevitably result in a sub-optimal research development process and reduce opportunities to generate meaningful research which underserves the needs of patients and/or the public.

## PPI recommendations in doctoral research within a sensitive subject

To support doctoral students engaging with PPI who are potentially working with bereaved parents and carers we have produced the following recommendations:

- *Enable ownership and a partnership approach from the outset through activities such as study branding* This involvement helped legitimise the study within the parent and healthcare professional communities. Some participants had heard of the study or seen the study logo which gave them confidence to participate creating authenticity and credibility of the study.
- *Maximise PPI engagement through an individualised approach to reduce emotional burden of participation* such as plan meeting to avoid significant dates, anniversaries, and holidays such as Christmas which can be an intense time for parents and carers.
- *Allow sufficient time for PPI members to review documents in advance of meetings* This reduced the potential for being overburdened, enabling time for members to review and process materials providing thought-through cohesive feedback particularly in relation to recruitment and data analysis. Parents and carers felt valued in having this time which maximised their engagement and supported the development of a collaborative partnership.
- *Provide regular study updates for ongoing connection and engagement with the work* This is important between group PPI meetings and 1:1 work with group members and plays an important role in continuing to strengthen shared ownership and how their involvement has informed and shaped the research study. Sharing updates also provides opportunity for PPI members to suggest alternative approaches or consider an aspect from a different viewpoint which has not been considered such as additional recruitment strategies.
- *Engage a clinical practitioner to support and safeguard parents’ and carers’ emotional and psychological well-being* Although this role developed, group members spoke of the positive benefit of having an additional person in the group who provided objectivity during discussions and added an additional perspective. The addition of the clinical nurse helped instil confidence among PPI members that their input was being purposefully discussed and considered, leading to them feeling valued and heard.
- *Ensure adequate funding to facilitate PPI throughout the research study* Funding is an essential requirement to engage and involve patients and the public and should be considered at the study outset. Depending on how and if the research is funded, consideration might need to be given to sourcing funding for example from NHS Trusts, Higher Education Institute budgets or approaching charities relevant to the study population. Sufficient funding to reimburse PPI activities needs to be factored into applications where funders do cover PPI costs particularly as it is difficult at the outset to foresee how these activities might develop throughout the study.

Final reflections from PPI group members on their involvement highlight the importance of sharing the PPI experience with others and the critical importance of developing plans for PPI from the very beginning of a research study.

“Working on this study has been a rewarding experience, and I hope this approach to PPI serves as a model for others.”

“It was quite a challenging experience overall. We learnt a lot about the process and the technical aspects of this type of study. It’s certainly not for the faint-hearted (we absolutely weren’t!) so an insight into what the work involves, and the level of engagement required at the outset for all participants, is key.”

## Strengths and limitations

There were concerns from academics and researchers that parent recruitment in the study might be a limitation. The PPI voice had a positive effect within the parent community increasing awareness of the study which resulted in some parents who participated having heard of the study prior to being approached which increased recruitment. This reinforces the definition of PPI carrying out research “with” members of the public as opposed “to”, “about”, or “for” them [1].

There was a lack of diversity within the PPI group which might be attributed to the small parent community. Two parents were active voices working within the charity sector with extensive knowledge of the disease and treatment landscape. Group members were all bereaved at the time the core PPI group convened which had the potential for recall bias and a different reflection of their experiences. However, the group recognised the limitations of parents and carers participating in a PPI group such as this when their child was receiving treatment. Caring responsibilities, uncertainty of their child’s outcome and emotional load of making these treatment decisions will have impacted their abilities to be involved should they have wished too.

There were times when suggestions by PPI members could be not incorporated into the study due to practicalities or details within the research protocol. For example, adding in additional hospitals to support recruitment which would have duplicated potential participants through hospitals which were already sites for the study. Each suggestion was always considered, discussed and if not feasible, explanation was given as to reasons why. This feedback ensured PPI members knew their voices had been listened to and heard with reasoning why some suggestions could not be actioned.

## Conclusion

This paper contributes to a growing knowledge-base on the approaches and application of PPI in doctoral research. Effective PPI is an essential component of doctoral studies to conduct meaningful and impactful research that addresses specific needs within a target population. The lead author received NIHR funding to complete this research and was professionally situated to engage with this parent population. This impacted how effectively PPI was engaged with, by having the resources (time, finance, existing relationships) to develop a PPI group and create a partnership approach over the six years of the study. For doctoral students without these resources there are PPI funding streams available for example through the NIHR Research Support Service for researchers to access to support initial PPI consultation work. Undertaking research in a community that the researcher is not involved in may require approaching those working with that population to support access and ideas to incorporate PPI. Patient and Public Involvement should not be considered as an ‘add-on’ or token contribution that is ‘nice-to-have’, but integral to the end-to-end research process.

A partnership approach fostering co-creation throughout the research cycle contributes to the best research outcomes and represents an opportunity for shared learning and effective collaboration. Evaluating PPI impact through group member reflections helped identify the components of effective PPI and what can be learnt to enhance this in the future. Doctoral students have the opportunity to learn in collaboration with their PPI groups to build confidence and nurture this partnership approach and in doing so has the potential to develop meaningful and impactful research.

### Supplementary Information

Additional file 1: GRIPP2 Short Form Checklist

Additional file 2: Parent Feedback on Study Design

## Data Availability

Findings from this research study are being published. The dataset analysed during the study are available from the corresponding author on reasonable request.

## Abbreviations

CCLG: Children’s Cancer Leukaemia Group
MRC: Medical Research Council
NIHR: National Institute for Health and Care Research
PPI: Patient public involvement
RSS: Research support service
UK: United Kingdom
