# Supplementary material for: Integration of patient and public involvement in a doctoral research study using the research cycle
Source: Res Involv Engagem. 2024 Aug 9;10:87. doi: 10.1186/s40900-024-00620-z (PMC11316368; doi:10.1186/s40900-024-00620-z)
Supplement: Supplementary file 1 — Additional file 1: GRIPP2 Short Form Checklist [file 40900_2024_620_MOESM1_ESM.docx]

**Additional File 1: GRIPP2 Short Form Checklist**

| **Section and Topic** | **Item** | **Reported on Page No** |
| --- | --- | --- |
| Aim | Report the aim of PPI in the study | 10-11 |
| Methods | Provide a clear description on the methods used in the study | 11-14 |
| Study results | Outcomes: Report the results of PPI in the study, including both the positive and negative outcomes | 15-21 |
| Discussion and conclusion | Outcomes: Comment on the extent to which PPI influenced the study overall. Describe positive and negative effects | 26-31 |
| Reflections/critical perspective | Comment critically on the study, reflecting on the things that went well and those that did not, so others can learn from this experience | 21-26 |
