# Supplementary material for: Integration of patient and public involvement in a doctoral research study using the research cycle
Source: Res Involv Engagem. 2024 Aug 9;10:87. doi: 10.1186/s40900-024-00620-z (PMC11316368; doi:10.1186/s40900-024-00620-z)
Supplement: Supplementary file 2 — Additional file 2: Parent Feedback on Study Design [file 40900_2024_620_MOESM2_ESM.docx]

**Additional file 2:: Initial meeting parent feedback on the study design**

| **Study Design Topics** | **Parent Feedback** | **How feedback incorporated into the study** |
| --- | --- | --- |
| What are the barriers and enables for parents to participate in research regarding decision making whilst their child is undergoing treatment? | - Need to be clear what the purpose of the research is and if there will be any benefit to participating. - Not to be clear the research does not help parents to make a decision. - Having the opportunity to talk to someone removed from their situation may help parents to clarify their thought processes ie: could be a positive experience for them. - Recruitment and/or withdrawal rate could be high due to the sensitivity of the topic. | Parent information sheet to provide clear information on the purpose of the research, why this is important and what it is trying to achieve.  Clear statement that there is not any direct benefit to participating but the findings from the study could help future parents with their decision-making.  Parents to be approached each time they make a treatment decision to see if they are willing to participate over time and not for a longitudinal study. |
| Suggested interview questions to incorporate into the interview schedule | - Consider questions in relation to how parents researched and found information on treatment options. - How did they decide on a particular treatment decision over the others? - What influenced their decision-making? Ie: other parents, family. - What helped parents feel supported in their decision-making? | Broad open-ended questions to be included in the interview schedule as follows:  Talk me through the latest treatment decision you have made?  What was important to you when making that decision?  What influenced you in making that decision? |
| Consideration of interview location (hospital, home, neutral) | - Parent to decide on when and where to be interviewed. - Parent to advise which method they would prefer (face-to-face, telephone). | Parent information sheet to offer flexibility in in interview location, time of the interview and how parents would prefer to do this. If parents are interested in participating these details can then be arranged with the researcher. |
| Following the interview would you show the interview transcript to parents for review/checking or if being reinterviewed would you show the previous interview transcript to refresh their memory? | - Could induce emotional reaction if parents read their interview transcript after the outcome of the treatment given and their child has deteriorated. - Seeing previous interview transcript may influence current decision making and introduce bias to the interview. - Parents are trying to move forward and have to live with previous decisions they have made so this would not be in the parents’ best interests. | Not to include interview transcript review/check for parents to minimise burden of participation in the study. |
| Ideas on how parents could be recruited for the study | - A member of the healthcare team who knows the family could approach parents regarding the study. - Having information from a healthcare professional parents know and trust may support engagement with the study. - Recruitment via social media ie: Twitter and Facebook which charities can share to increase awareness and engagement with the parent community although potential for parents not to engage if they have not been personally spoken to as a sensitive topic. | To start with recruitment via the Primary Treatment Centres (PTC) for childhood cancer in the UK.  See what engagement there is via this route before considering recruitment via social media. |
| Consideration on parents that should be included or excluded from the study | - Not ethical to recruit at the point parents are told their child has relapsed/refractory disease, study needs to be introduced once a parent has made a treatment decision and before the outcome is known so they are now biased by the treatment outcome. - The burden of decision-making is difficult for parents therefore focus on parents’ whose English is their first language. - Parents need to have capacity to consent. | Inclusion criteria:  Parents to have made the treatment decision and then be approached regarding the study.  Exclusion criteria:  Parents whose English is not their first language.  Healthcare professionals providing information regarding the study who deem it inappropriate to be included due to psychological concerns ie: mental health, child’s clinical state. |
| Ways to minimise burden to parents who participate in the study | - Important not to be intrusive when contacting parents to participate and onus for contacting parents should be on the researcher. Suggested to make contact twice and if no response assume they have declined participation. - Do not discuss regret directly unless parents bring this up themselves. - Do not ask ‘if you would have done something differently’. - Sensitivity of the use of language during the interview. | The PTC to share parent contact details with the researcher to enable them to contact the parents to discuss the study/organise an interview.  To make contact twice and if no response to discontinue.  Interview questions to be drafted and reviewed by the PPI group for input and amendments.  Researcher to attend relevant qualitative training to undertake interviews. |
| What are parent concerns regarding participation in the study? | - Concern re interview times – childcare/work during daytime, option of evening and weekend interviews. - This is an important area to research, parents are likely to welcome the opportunity to talk about their experiences. | Researcher to offer flexible times for interviews including evening and weekends. |
